# AIDS-related progressive multifocal leukoencephalopathy

**DOI:** 10.1590/0037-8682-0252-2020

**Published:** 2020-12-21

**Authors:** Carolina de Oliveira Abrão, Lissa Rodrigues Machado da Silva, Luiz Carlos Silva Souza, Nathalia de Mello Bisso, Marília Dalva Turchi, Adriana Oliveira Guilarde

**Affiliations:** 1 Secretaria de Estado da Saúde de Goiás, Hospital de Doenças Tropicais Dr. Anuar Auad, Goiânia, GO, Brasil.; 2 Universidade Federal de Goiás, Instituto de Patologia Tropical e Saúde Pública, Goiânia, GO, Brasil.

**Keywords:** Progressive multifocal leukoencephalopathy, JC polyomavirus encephalopathy, JC virus

## Abstract

**INTRODUCTION:**

*Progressive multifocal leukoencephalopathy (PML) is a* demyelinating disease of the central nervous system caused by reactivation of *JC virus* (JCV).

**METHODS:**

We described the profile of laboratory-confirmed PML cases among AIDS patients.

**RESULTS:**

*A total of 43 HIV patients with* clinical conditions compatible with PML were obtained; 5 cases were confirmed by JCV testing. The main clinical finding was mental confusion. Median CD4 count was 54 cells/mm³.

**CONCLUSIONS:**

Three of the five confirmed PML cases died; the time between diagnosis and death was 2, 5, and 6 months. It is important to consider JCV infection as a differential diagnosis.

Progressive multifocal leukoencephalopathy (PML) is a demyelinating disease of the central nervous system (CNS) caused by reactivation of *John Cunningham Virus* (JCV), a double-stranded, non-enveloped, DNA virus of the Polyomaviridae family, that was recently renamed Polyomavirus JC (JCPyV)^1^ ^,^ ^2^. It is neurotropic, omnipresent in nature, and species-specific, being found only in humans. Thus, research on the pathogenesis of the virus has been hindered by the lack of an animal model. The virus has a global distribution, and 40-86% of the population is estimated to be infected. Primary infection occurs in the first two decades of life and is asymptomatic in most cases. The virus then remains latent in the kidneys. In fact, it can be detected in the urine of 10-30% of individuals, healthy or immunodeficient, with or without PML. Approximately 39% of individuals have a positive PCR test in tonsil tissues, which suggests a possible oral or respiratory route of infection, but the mode of transmission is not well established. The presence of the virus in the bone marrow and brain of PML patients indicates that they are also possible areas of latency. However, JCV viremia is normally detected only in immunodeficient patients, and in 20-40% of HIV patients without PML and 60-80% of patients with PML^3^ ^,^ ^4^.

HIV is the main predisposing condition for the development of the disease, becoming more common than other causes of immunosuppression, and the incidence increased 50 times. Currently, 82% of PML patients have HIV. It is recognized as an important opportunistic infection that affects up to 5% of untreated HIV patients and is classified as an AIDS-defining opportunistic infection. In Brazil, it’s frequency is not well established, but it is the fourth most frequent neurologic opportunistic complication, after neurotoxoplasmosis, neurocryptococcosis and neurotuberculosis^5^. In large centers, even in the era of highly active antiretroviral therapy (HAART), cases have been described, especially in association with immune reconstitution inflammatory syndrome (IRIS)^6^.

This study aimed to describe the clinical and laboratory profile of laboratory-confirmed PML cases among AIDS patients in a reference center in Brazil.

A case series of adult HIV-positive patients with neuroinfection, hospitalized in an infectious diseases reference hospital in Goias, from March, 2013 to March, 2015 was included in the study. Initially, a search was made within all the laboratory records for JCV testing in the cerebrospinal fluid (CSF) of patients with HIV suspected to have PML. These included patients with muscle weakness, sensory deficit, hemianopsia, cognitive dysfunction, aphasia, and/or coordination and gait difficulties^7^. The period was determined from when the institution’s laboratory began providing molecular testing for JCV (March, 2013).

JCV testing in CSF was performed by a FRET real-time PCR assay^8^. CD4+ T cell count was performed using a Multitest Facscalibur flow cytometer, and HIV viral load was measured by bDNA assay up until April 2013, and by Abbott real-time HIV-1 assay as of May 2013.

The reviewed medical records included age, sex, clinical symptoms, CD4+ T cell count, HIV viral load, ART, and results of the computed tomography (CT) and/or magnetic resonance imaging (MRI).

A total of 45 HIV-infected patients with clinical conditions compatible with PML were obtained. Two cases were excluded because they were duplicates, and the same patient was admitted more than once. Within the suspected cases, five were confirmed by positive JCV testing in CSF. Age varied from 46 to 62 years, with a mean of 51 years (standard deviation [SD]: 1.3 years), and 60% (3) were male (Table 1). The clinical manifestations were mental confusion and altered state of consciousness (2), gait disorders and motor deficits (2), and amaurosis and mental confusion (1). Three patients were diagnosed with HIV upon hospital admission and two had been diagnosed 6 and 8 years previously, both having abandoned treatment. Median CD4+ T cell count was 54 cells/mm³, ranging from 6-130-cells/mm³. Median HIV viral load was 91,984 copies/mL (range: 1,469-2,647,418).

**TABLE 1:** PML cases in an infectious diseases reference hospital in Goias, Brazil, from March, 2013 to March, 2015.

Case	Age (years)	Symptoms	CD4+ (cells/mm^3^)	ART	Imaging tests	Outcome
1	62	Mental confusion	65	AZT/3TC + LPV/r	CT - Multiple foci of asymmetric cortico-subcortical hypodensities in both cerebral and cerebellar hemispheres	Death
2	48	Mental confusion Amaurosis	06	AZT/3TC + TDF + LPV/r	MRI - Lesion in the centrum semiovale, periventricular and subcortical white matter of the occipital and right temporal lobes, in addition to involvement of the splenium of the corpus callosum.	Death
3	46	Psychomotor agitation	54	TDF + 3TC + EFV	CT - Cortico-subcortical hypoattenuation in the posterior aspect of the left cerebellar hemisphere	Discharged
4	62	Dizziness, Mental confusion Paresis of both legs	54	No ART	MRI **-** Foci of signal alterations of the splenium of the corpus callosum bilaterally, left frontoparietal white matter, right frontal high convexity, and left cerebellar hemisphere.	Death
5	57	Right hemiplegia	130	TDF + 3TC + EFV	MRI **-** Extensive area of confluent signal alteration compromising part of the left parieto-occipital transition area and the left occipital lobe, as well as the right precentral gyrus (less evidently), without mass effect or intravenous contrast impregnation	Discharged

**PML:** progressive multifocal leukoencephalopathy; **ART:** antiretroviral therapy; **AZT:** zidovudine; **3TC:** lamivudine; **LPV:** lopinavir; **r:** ritonavir; **TDF:** tenofovir; **EFV:** efavirenz; **CT:** computed tomography; **MRI:** magnetic resonance imaging.

All of the confirmed cases underwent a head CT upon admission, which showed hypodensity areas of the white matter in three cases, the parieto-occipital region in one case, and the temporo-parietal region in two cases. Multifocal lesions occurred in four cases. Two patients had cerebellar lesions. Amongst all cases, three underwent a head contrasted MRI, which showed lesions compatible with the patients’ CT scans. The main alterations were confluent signal alteration compromising part of the left parieto-occipital transition area and the left occipital lobe, as well as the right precentral gyrus, with no mass effect or intravenous contrast impregnation. In both cases, bilateral signal alteration of the splenium of the corpus callosum and of the frontoparietal white matter were observed.

The above data concerning the five confirmed PML cases are summarized in Table 1. Table 2 describes the features of the suspected, non-confirmed cases. Specific tests were performed to investigate other differential diagnoses, such as: cryptococcal antigen, fungal direct smear, fungal culture, and polymerase chain reaction for tuberculosis, bacterial, and mycobacterial cultures.

**TABLE 2:** Features of suspected, non confirmed cases of PML (n = 43).

Sex Male (%)	Age-years Mean (sd)	Differential Diagnosis N (%)	Median CD4+(min.- max.)	Median Viral Load (min.- max.)	Death (%)
28 (65.1)	40 (10.0)	Neurotoxoplasmosis 21 (48.8)	39 (1-905)	157,968 (undetectable-5,620,605)	14 (32.5)
		Neurocryptococcosis 7 (16.3)			
		Neurotuberculosis 5 (11.6)			
		Others 10 (23.2)			

**PML:** progressive multifocal leukoencephalopathy; **CD4+:** CD4 lymphocyte count.

Only two confirmed PML patients showed abnormal CSF analysis results, with a pleocytosis of 20 and 288 leukocytes, lymphomononuclear predominance, and one case showed hyperproteinorrhachia and hypoglycorrhachia.

Three of the five confirmed cases died. All deaths occurred in patients with a recent diagnosis of HIV, with a CD4+ T cell count of less than 65 and a viral load of more than 1,469 copies/mL. The time between diagnosis and death was 2 months, 5 months, and 6 months. The lethality rate observed in the whole sample of suspected cases was 32.5% (14/43).

Our study demonstrates the relevance of JCV infection in patients living with HIV/AIDS. A retrospective Brazilian study on HIV patients and CNS infection by JCV demonstrated a mean CD4+ T cell count of 65 cells/mm³, with a count of < 100 cells/mm³ being associated with a worse outcome. In our case series, the patients who died had a CD4+ T cell count of ≤ 65 cells/mm³. Incidence rates of 0.2 and 9.1/1,000 people per year at risk, for patients with a CD4+ T cell counts ≥ 200 versus < 200 cells/mm³ have been described. The estimated survival after one year is 48% in HIV patients with a CD4+ T cell count < 200 upon diagnosis, compared to 67% in those with a CD4+ T cell count > 200 cells/mm³. A worse prognosis is also observed in patients with a previous diagnosis of HIV infection, probably because this reflects a low adherence to ART or virus resistence^6^. In our study, three patients had a recent diagnosis of HIV infection when PML was diagnosed; other authors have described PML as the first manifestation of immunodeficiency, but this is not usually the initial presentation of the disease^9^. A definitive diagnosis is given upon detection of JCV in CSF using PCR, or in cerebral tissues through biopsy. A possible diagnosis is made when imaging exams and clinical manifestations are compatible in the absence of virus detection^2^ ^,^ ^3^. We found 43 possible cases, but virus detection positivity was low at 11.1% (5/43).

Radiographically, PML cerebral lesions appear as multiple white matter lesions, sparing the cortex, and frequently located in the subcortical region of the cerebral hemispheres and cerebellar peduncles^1^ ^,^ ^2^. As the virus disseminates from cell to cell, each focus increases. The disease is bilateral, but asymmetrical, but it can be unilateral and have one single lesion. The parietal lobe is most commonly affected, followed by the frontal lobe. The lesions appear as hypodensities in the white matter on CT, and as T2 and FLAIR hyperintensities and T1 hypodensities on MRI. MRI is more sensitive than CT and is the first choice for the diagnosis of MPL^10^. In our cases, the main alterations were white matter lesions, without evidence of mass effect or contrast impregnation.

The percentage of cases confirmed by JCV testing in CSF of patients with a clinical condition compatible with PML was 11.1%. The main clinical finding was mental confusion. It is important to consider JCV infection as a differential diagnosis in these cases, which are often sub notified due to limitations in diagnostic tools. 
